# Photon Counting Imaging with Low Noise and a Wide Dynamic Range for Aurora Observations

**DOI:** 10.3390/s20205958

**Published:** 2020-10-21

**Authors:** Zhen-Wei Han, Ke-Fei Song, Hong-Ji Zhang, Miao Yu, Ling-Ping He, Quan-Feng Guo, Xue Wang, Yang Liu, Bo Chen

**Affiliations:** 1 Changchun Institute of Optics, Fine Mechanics and Physics, Chinese Academy of Sciences, Changchun 130033, China; hanzw@ciomp.ac.cn (Z.-W.H.); songkf@ciomp.ac.cn (K.-F.S.); zhanghj@ciomp.ac.cn (H.-J.Z.); help@ciomp.ac.cn (L.-P.H.); guoqf@ciomp.ac.cn (Q.-F.G.); snow85116@163.com (X.W.); liuy_ciomp@163.com (Y.L.); 2 Daheng School, University of Chinese Academy of Sciences, Beijing 100049, China; 3 Shanghai Institute of Satellite Engineering, Shanghai 200240, China; 32050425yumiao@sina.com

**Keywords:** photon counting imaging, far ultraviolet (FUV) instrument, microchannel plate (MCP), readout anode, charge sensitive amplifier (CSA), pulse shaper

## Abstract

The radiation intensity of observed auroras in the far-ultraviolet (FUV) band varies dramatically with location for aerospace applications, requiring a photon counting imaging apparatus with a wide dynamic range. However, combining high spatial resolution imaging with high event rates is technically challenging. We developed an FUV photon counting imaging system for aurora observation. Our system mainly consists of a microchannel plate (MCP) stack readout using a wedge strip anode (WSA) with charge induction and high-speed electronics, such as a charge sensitive amplifier (CSA) and pulse shaper. Moreover, we constructed an anode readout model and a time response model for readout circuits to investigate the counting error in high counting rate applications. This system supports global rates of 500 kilo counts, 0.610 dark counts s^−1^ cm^−2^ at an ambient temperature of 300 K and 111 µm spatial resolution at 400 kilo counts s^−1^ (kcps). We demonstrate an obvious photon count loss at incident intensities close to the counting capacity of the system. To preserve image quality, the response time should be improved and some noise performance may be sacrificed. Finally, we also describe the correlation between counting rate and imaging resolution, which further guides the design of space observation instruments.

## 1. Introduction

Auroras are natural light displays in the Earth’s sky, which are predominantly observed in the high-latitude regions around the Arctic and Antarctic, resulting from disturbances in the magnetosphere caused by solar wind [1,2]. Geomagnetic activities have been shown to be closely correlated with the intensity and distribution of auroras. Thus, aurora research elucidates the disturbance of the Earth’s magnetic field during magnetic storms and substorms, the magnetospheric plasma injection process, the ionospheric response to solar wind changes and the dynamic characteristics of ionosphere–magnetosphere interactions [3,4,5]. Our team developed the extreme ultraviolet camera (EUVC) for the lunar lander of the Chang’e-III mission, launched in 2013, to capture global and instantaneous EUV images of the Earth’s plasmasphere [6,7]. To further obtain unambiguous identification of the particles giving rise to the aurora, we also designed the wide-angle aurora imager (WAI) for the low polar orbit’s FY3 satellite mission. This imager has been monitoring the all-weather activity of the Earth’s ionosphere since 2018 [8,9].

The instruments typically operate in the FUV wavelength range from 140 to 180 nm, which contains molecular N_2_ Lyman–Birge–Hopfield (LBH) emissions [8,10]. The intensity of LBH aurora radiation in polar dayglow conditions is usually greater than 10 k Rayleigh. Meanwhile, the intensity in Earth’s polar caps can be as low as 100 Rayleigh [8,11]. To accurately obtain the aurora morphological distribution over time and space, the detection system must accommodate not only intense radiation, but also weak signals with a wide dynamic response [12]. According to the requirements for the specific scientific objectives, the main index of the WAI instrument is designed. For instance, the field of view is 130° (cross-track) × 10° (along-track), angular resolution is 0.8° (10 km at the nadir in disk images) and sensitivity is 0.01 counts s^−1^ Rayleigh^−1^ pixel^−1^ [8]. See [8] for more detailed parameters. For a 30,000-Rayleigh emission, one pixel of the detector can receive 300 photons in 1 s. All pixels in the window area of the detector will be filled with photoelectrons during magnetic storms. The maximum event rates for the detector would reach 331 kcps. Thus, photon counting imaging based on MCP is considered to be superior with respect to this performance. This is obvious at very low fluxes, where low noise and high sensitivity are crucial for the photon counting system. However, photon counting imaging also exhibits a speed advantage in detection at high fluxes. For an unsuitable system, the probability of particle event pile-up will increase at high incident event rates, which leads to deterioration of image quality.

An MCP sense wire (MCP-SW) detector using a delay line readout has been utilized to achieve a spatial resolution of approximately 200 µm FWHM [13,14]. However, the manufacturing process is relatively complex and improved material properties are required. The cross-strip anode as an MCP readout has also been developed to obtain event rates up to 300 kcps or even Mcps [15]. The main difficulty is the need to provide a readout circuit for each of the strips, which requires high power consumption and spatial size. Although application specific integrated circuits (ASICs) have been applied to lower the power and volume requirements in recent years [16], the cost of ASICs qualified for flight is significant. ASICs usually take a long time to develop. These approaches may not be suitable for the resolution that this specific application requires. A simple and compact architecture should be considered for the flight version of the instrument.

This work’s main objective was to implement a low-noise photon counting imaging system with a wide dynamic range to facilitate the observation of aurora intensity for aerospace applications. In addition to the MCP detector’s design details, we also evaluated the inherent noise, response time and linearity of the counting rates for readout circuits, including the CSA and pulse shaper. To understand the factors that limit the measured resolution, the effects of incident event rates on the imaging system’s spatial resolution were further discussed. Our research has demonstrated that the system is feasible and can achieve decent counting rates and imaging resolution. At the end of the paper, we provide an outlook on a route for future improvements and one possible solution for shortcomings.

## 2. Design of the Detection System with Photon Counting

The incident photons from faint objects appear as a series of single photons irradiating the detector’s surface, which can be converted by the detector into several charge pulses. These charge pulses are subsequently transmitted to the readout circuits, which determine the electron cloud’s central location; that is, the coordinates of the incident photons. After accumulation for a certain period of time, it is possible to construct two-dimensional images indicating the intensity and outline of aerospace objects [12,17].

### 2.1. The Structure of Detector Assemblies

**Figure 1:**
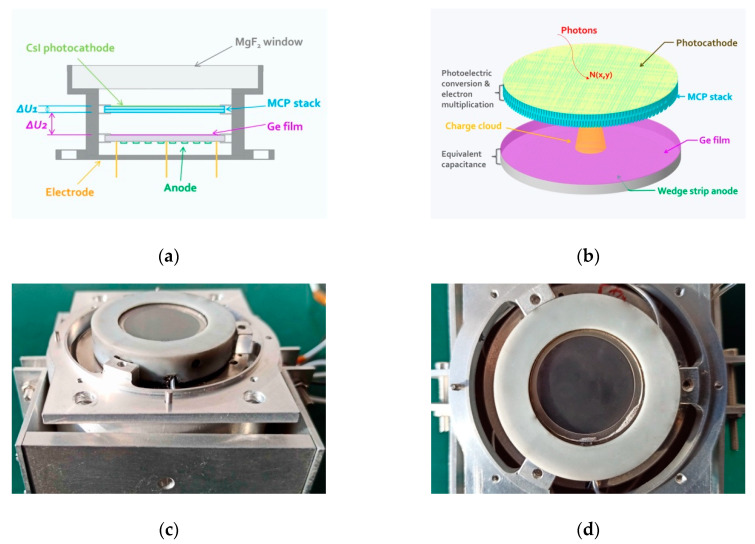
Schematic diagram and photograph of the FUV imaging detector. (**a**) Components and structure of the detector. A photoelectron is converted from a photon through the photocathode with CsI. Then, the photoelectron enters a pore of the MCP and collides with the wall to produce secondary electrons. A potential of Δ*U*_1_ across the two MCPs accelerates these electrons through angled capillaries, producing an electron cloud. A final potential of Δ*U*_2_ guides this electron cloud to the germanium film for readouts. Here, Δ*U*_1_ and Δ*U*_2_ indicate the applied voltage differences from the high-voltage divider. (**b**) Schematic of the detector with induction readout mode. The electron cloud emanating from the MCPs can be coupled to the anode through the equivalent capacitance and finally is released through the germanium film. (**c**,**d**) Photographs of the FUV imaging detector with a wedge strip anode. In addition to providing a vacuum environment for the function of the MCPs, the enclosed cavity acts as an electromagnetic shield.

We developed a single-photon imaging detector in the FUV band, the structure of which is depicted in Figure 1. An entrance window with magnesium fluoride (MgF_2_) in the front end allows incident photons to enter the detector. These photons are further converted into photoelectrons through the photocathode with cesium iodide (CsI), which is plated on the MCP surface to achieve high quantum efficiency at wavelengths of 140–180 nm. The maximum quantum efficiency of the photocathode is approximately 12.9% at a wavelength of 140 nm. Afterwards, photoelectrons produced at the photocathode are accelerated towards the MCP stack as a result of the voltage applied between the two.

A pair of MCPs was designed as a chevron stack that was closely placed back-to-back with a diameter of 33 mm (active area of 25 mm diameter), 12.5 µm diameter channels, a length-to-diameter ratio of 40:1, and a total stack resistance of 150 MΩ. Electrons striking the MCPs usually give rise to an avalanche of secondary electrons with a certain probability. Thus, the front and rear MCPs maintain a 6 degree bias (i.e., the angle between the channel axis and the normal to the surface of the plate) to ensure that sufficient secondary electrons are generated (approximately 10^7^–10^8^ electrons).

Behind the MCP stack, charge collection and induction devices are installed with a separation of 5 mm. The germanium film with a thickness of 100 nm and selected anode constitute a capacitive coupling with the fused silica with a thickness of 2 mm [18]. The electron clouds produced in the pores of the MCPs are all collected on the film, which serves to localize the event while allowing discharge over a longer time period. Accordingly, the centroid position of the incident photon can be extrapolated by measuring the deposited charge distribution on the anode for each event. The centroid position (*X*, *Y*) can be calculated by Equation (1). All components mentioned above are sealed in a vacuum chamber and its housing is used as a ground potential for the charge output and power supply of the detector.

(1) $$\begin{array}{l} X=K\times a_{1}\frac{Q_{S}}{Q_{S}+Q_{W}+Q_{Z}}-x_{0}\\ Y=K\times a_{2}\frac{Q_{W}}{Q_{S}+Q_{W}+Q_{Z}}-y_{0} \end{array}$$

where *Q_S_, Q_W_* and *Q_Z_* are the charge collected on the wedge, strip and zigzag electrodes, respectively; the parameters *K*, *a*_1_ and *a*_2_ are scale FACTORS; and *x*_0_ and *y*_0_ are shift factors.

**Figure 2:**
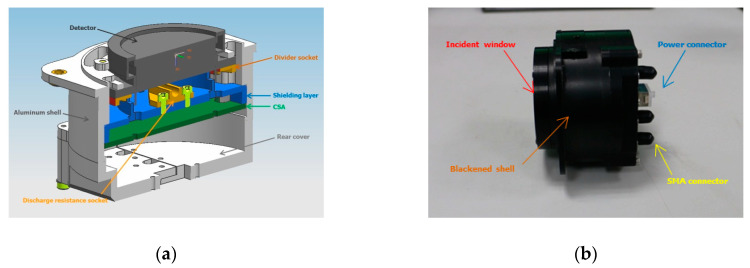
(**a**) Profile of an FUV imaging detector module. It is composed of an MCP detector with a wedge strip anode, a high-voltage divider, and three charge-sensitive spectroscopy amplifiers. (**b**) Photograph of an FUV imaging detector. The aluminum shell was anodized in black to satisfy the requirements of thermal control. Three-channel pulse signals from CSAs travel down pulse shapers through the SMA connectors.

The detector assembly is further illustrated in Figure 2. The high-voltage divider, which provides the bias voltage to the MCPs, is installed outside the MCP detector’s vacuum chamber. To avoid electric discharge in the vacuum, this high-voltage divider is fixed in the Kapton socket and kept some distance from the sidewalls. A 1 mm thick aluminum shield is inserted between the MCP detector and the CSA to reduce electromagnetic interference. Three CSAs are compactly placed on the printed circuit board (PCB) near the shield layer. The distance between the MCP detector and the CSA is less than 20 mm to reduce additional noise during the propagation of charge pulses. In addition, the high-voltage divider can be supplied via a coaxial connector, which typically withstands a voltage of 5000 V for derating. Likewise, the signal outputs of three CSAs are carried out through coaxial connectors for high frequency. All of the components (an MCP detector, a high-voltage divider and a PCB) are assembled in a cylindrical aluminum housing with a diameter of 100 mm. In our previous work, the detector was not sealed in a vacuum chamber and could not be tested flexibly in the atmosphere. Moreover, the long distance between the detector and the CSA through the coaxial cable is not conducive to charge signal transmission. The improved structure is more compact, and the anti-interference ability is strengthened in the electric field radiation sensitivity experiment (frequency range 10 k–40 GHz, radiation electric field 20 V/m).

### 2.2. Evaluation of Readout Modes with Charge Induction

**Figure 3:**
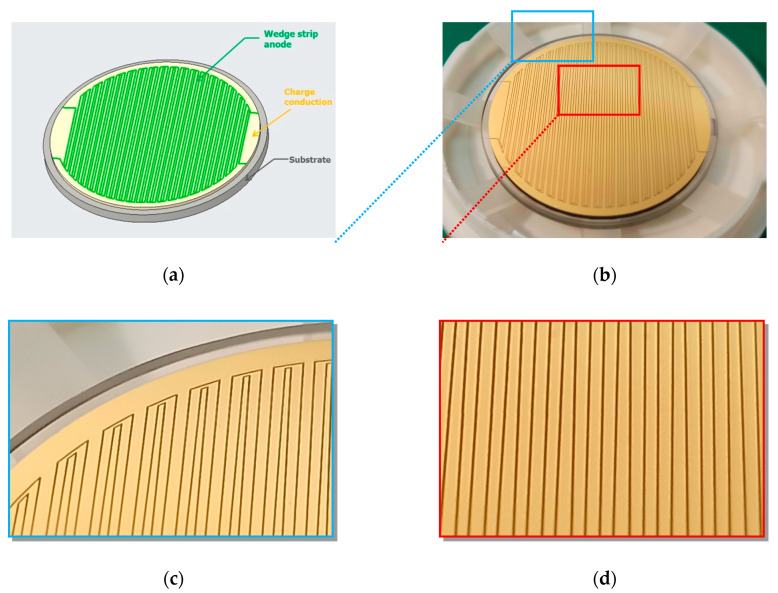
A pattern of a wedge strip anode for induced charge. (**a**) Model of a wedge strip anode. (**b**) Photograph of a wedge strip anode; (**c**) Zoomed details from the blue dotted box at the top left of (**b**). (**d**) Zoomed details from the red dotted box in the central area of (**b**).

The single event time response of MCP has been reported to be in the range of 100 ps [19]. This property has been extensively applied in high incident event rate applications to obtain high temporal resolution. We expected a somewhat degraded time response because the transit time spread from the MCPs to the germanium film. Figure 1b depicts the induction readout mode, in which the induced charge can be attained on the anode through the equivalent capacitance between the germanium film and its dielectric substrate. The wedge strip anode was made by selecting a material with a small dielectric constant as the substrate, as shown in Figure 3. We employed a picosecond laser to remove the aluminum film on the silica surface and subsequently etched the anode pattern. The inter-electrode capacitance among each anode pad was approximately 40 pF, as expected.

**Figure 4:**
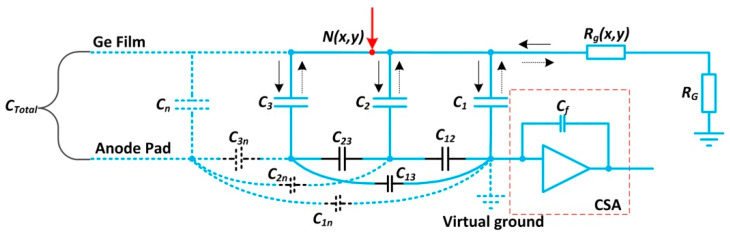
A lumped-parameter presentation of the readout mode for induced charge. *C*_1_, *C*_2_, *C*_3_ to *C_n_* = the capacitances between the germanium film and each anode pad, respectively. *C*_12_, *C*_23_, *C*_13_ to *C_1n_*, *C_2n_*, *C_3n_* = the inter-electrode capacitances for each anode pad. *C_Total_* = the capacitance between the germanium film and the virtual ground. *N(x,y)* represents the electron cloud position to be used in the calculation. *R_g_(x,y)* is the germanium film’s surface resistance between any node *N(x,y)* and the discharged resistance *R_G_*.

The readout mode can be viewed as an RC transmission line with the time constant *τ_rd_*. Here, *R* is the total discharged resistance and *C* is the total equivalent capacitance between the germanium film and the anode, which is usually dependent on the geometric configuration. Figure 4 illuminates the simple model that describes the process of capacitor charge or discharge during charge induction. *N(x,y)* indicates the node where the electron cloud lands on the germanium film. *C*_1_, *C*_2_ to *C_n_* represent the capacitances between the germanium film and each anode pad, respectively. The capacitor in the dotted line may exist in other types of readout anodes, such as the multi-anode and Vernier anode. Although the capacitances between the anode and reference points are usually ignored for the virtual ground node, the total capacitance between the germanium film and the reference points is dependent on the inter-electrode capacitances of charge division anodes in the black line in addition to the capacitances *C*_1_, *C*_2_ to *C_n_*. For the charge release pathway, *R_g_(x,y)* is used to evaluate the resistance. This represents the resistance between any node *N(x,y)* and the discharged resistance *R_G_* located on the germanium film’s edge. Additionally, the readout circuits are shown in the red dotted box, where only one CSA is presented as an example.

The direction of the electron flow is indicated by the solid arrow when the capacitors are charged. Similarly, the opposite direction illustrates that the capacitors are discharging. The time constant *τ_rd_,* in which each localized charge arriving at the germanium film is dispersed, can be expressed as:

(2) $$\tau_{rd}=\left(MAX\{R_{g}(x,y)\}+R_{G}\right)*C_{Total}$$

Given that *T_cr_* is the duration of the electron cloud at node *N(x,y)* and *T_w_* is the arriving interval of the two continuous events, the time constant *τ_rd_* needs to be limited to the following range:

(3) $$10*T_{cr}<\tau_{rd}<T_{w},$$

When *τ_rd_* is more than ten times greater than *T_cr_*, the charge collected by the germanium film can be fully coupled to the anode without any loss. Each charge pulse from the anode has approximately the same shape, retaining enough information for data analysis. In contrast, the charge on the germanium film can only be partially coupled to the anode, resulting in deterioration of the detector resolution regardless of the incident photon rates. On the other hand, the charge on the germanium film cannot be completely released when *τ_rd_* is more than or equal to *T_w_*. This effect can cause pulse pile-up when a subsequent charge is detected before previous photon events have decayed. An evaluation of the relation between *τ_rd_* and *T_w_* is significant in high incident photon rate applications.

Because the arriving interval of photon events conforms to the Poisson distribution [17,20], the probability of *N* photoelectric pulses emanating from MCPs in time *t* is derived:

(4) $$P(N,t)=\frac{{\left(R_{i}t\right)}^{N}\exp(-R_{i}t)}{N!}$$

where $R_{i}$ is the average counting rate of photons, taking into account quantum efficiency.

The time interval between any two adjacent pulses entering the ideal counter is not less than *τ_rd_*, and the counting error at the readout anode can, thus, be derived:

(5) $$\xi_{rd}=1-\exp(-R_{i}\tau_{rd})$$

To reduce the counting error in high counting rate applications, it is necessary to thoroughly analyze the response time of other units of the detection system and not only the readout link, as discussed in the following sections.

### 2.3. Response of Readout Circuits

**Figure 5:**
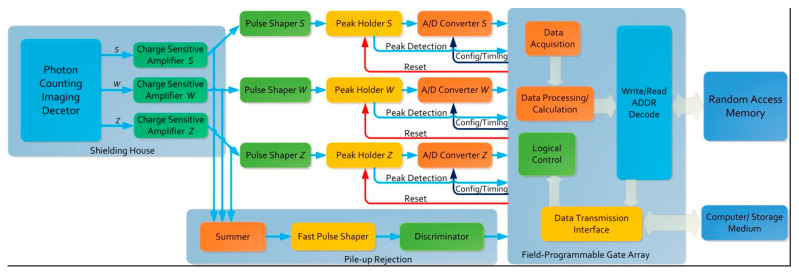
Block diagram of the readout circuits with the wedge strip anode. The labels *S*, *W* and *Z* in each module correspond to the channels of the strip electrode, wedge electrode and residual electrode, respectively. In practice, these three channels can be extended to more channels for other geometry configurations of anodes.

The photon counting technique’s performance for aurora detection also depends on the electronic system’s capacity to separate serial events [21]. The topology of readout circuits is essential in achieving a low-noise detection system with a wide dynamic range. Figure 5 provides an overview of the readout circuits’ overall architecture with the different modules involved, which was developed for space-borne observations considering the wedge strip anode in Figure 3. Each channel consists of one CSA encapsulated in the shielding house with a photon counting imaging detector followed by the pulse shaper, which is usually implemented with two-stage RC networks to form a quasi-Gaussian pulse, and finally the A/D converter with a peak holder. In addition, the pulse pile-up rejection component sums up output voltages from three CSAs and then compares them with the upper or lower threshold level. If the total pulse amplitude triggers the threshold voltage, the corresponding state signal can be set. According to the time interval of this state signal, we can determine whether pulse pile-up events occur. Therefore, a few events may be rejected when successive pulses occur close to each other. The operations of threshold regulation, data acquisition and calculation, and logical control are also performed in the field programmable gate array (FPGA).

The response time of readout circuits is primarily determined by the rise time of the CSA and the time constant of the pulse shaper; the response time, in turn, affects the linear and dynamic range of the detection system. During the whole photoelectric event, the electron cloud is swept into the electrodes and subsequently gives rise to a current pulse with a time duration in the order of nanoseconds. To minimize the possibility of current pulses overlapping, a fast response characteristic is usually required of the CSA. The CSA is an essential component in the front end of analogue signal processing chains [22].

**Figure 6:**
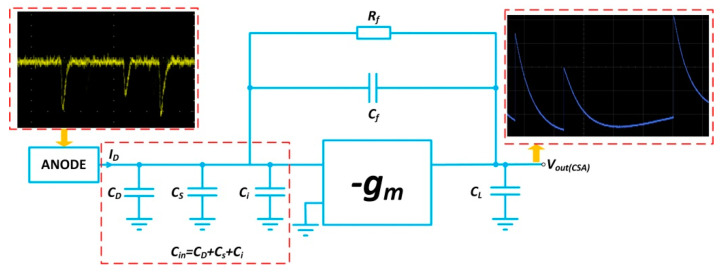
Schematic drawing of the CSA. The oscillogram in the red dotted box at the top left shows several narrow pulses of input current and long-tail pulses with a duration of *R_f_C_f_* in the red dotted box at the top right.

As illustrated in Figure 6, the CSA consists of an operational transconductance amplifier (OTA), a feedback resistor *R_f_*, a feedback capacitor *C_f_* and a load capacitance *C_L_* [12]. The MCP detector is modeled as a capacitor *C_D_* in parallel with the anode’s current pulse *I_D_*. The total capacitance *C_in_* on the OTA’s virtual ground node is approximately equal to the sum of the detector capacitance *C_D_*, stray capacitance *C_s_* and OTA input capacitance *C_i_*.

Once a pulse of induced charge from MCP output appears on the virtual ground node, the step voltage at the CSA output is created by the feedback capacitor *C_f_* as a result of the feedback operation. Afterwards, the charged capacitor *C_f_* can be reset by the feedback resistor *R_f_* until the next event arrives. An exponential decay follows the output voltage’s peak point, while the feedback resistor *R_f_* brings the CSA back to its steady-state.

**Figure 7:**
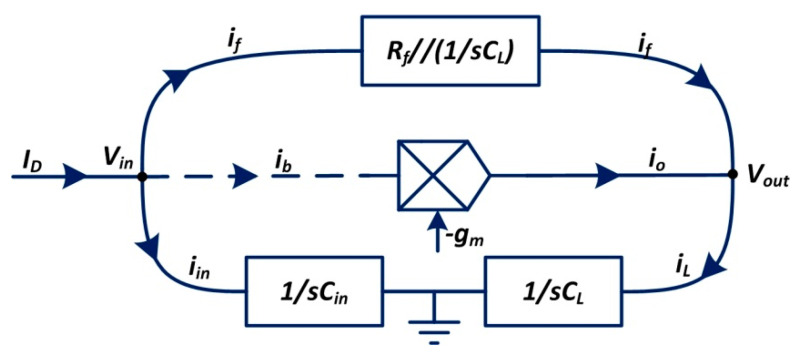
Current flow diagram of the CSA. The current *I_D_* from the MCP detector flows to the currents *i_f_* and *i_in_*. The current *i_b_* in the dotted line is typically ignored because of the OTA’s high input impedance characteristics. In the same way, the current *i_L_* amounts to the convergence of the currents *i_f_* and *i_o_*_._ Node *V_in_* and node *V_out_* are presented in the form of voltage.

The current flow is illustrated in Figure 7 to evaluate the characteristics of the CSA. The current *i_f_* flows through the feedback network, which determines the output voltage associated with the current *i_o_*. To achieve a high slew rate for the CSA’s output voltage, a sufficiently large *g_m_* is expected to generate a huge output current *i_o_*.

According to the Kirchhoff current law (KCL) and the Kirchhoff voltage law (KVL), we can derive:

(6) $$\left\{ \begin{array}{l} I_{D}=i_{in}+i_{f}\\ -i_{in}\cdot \frac{1}{sC_{in}}\cdot g_{m}=i_{o}\\ V_{out}=\left(i_{o}+i_{f}\right)\frac{1}{sC_{L}}\\ V_{out}+i_{f}\frac{R_{f}\frac{1}{sC_{f}}}{R_{f}+\frac{1}{sC_{f}}}=i_{in}\frac{1}{sC_{in}} \end{array}\right.$$

where *I_D_* is the anode current from the detector, *i_in_* is the current flowing into the total capacitance *C_in_* of the virtual ground node, *g_m_* stands for the transconductance value of the OTA and *i_L_* corresponds to the current flowing into the load capacitance *C_L_*.

The transimpedance function *H(s)_CSA_*, which establishes a connection between the CSA output voltage *V_out_* and the anode current *I_D_* from the detector, is given as follows:

(7) $$H{\left(s\right)}_{CSA}=\frac{R_{f}\left(1-s\frac{C_{f}}{g_{m}}\right)+\frac{1}{g_{m}}}{1+s\left(R_{f}C_{f}+\frac{c_{L}+c_{D}}{g_{m}}\right)+s^{2}\left(C_{L}C_{D}+C_{L}C_{f}+C_{D}C_{f}\right)\frac{R_{f}}{g_{m}}}$$

For sufficiently high transconductance *g_m_*, the *H(s)_CSA_*, which does not take into account *1/g_m_* and *(C_L_ + C_D_)/g_m_C_f_*, is given as follows:

(8) $$H{\left(s\right)}_{CSA}=\frac{R_{f}\left(1-s\frac{C_{f}}{g_{m}}\right)}{\left(1+sR_{f}C_{f}\right)\left(1+s\frac{\left(C_{L}C_{D}+C_{L}C_{f}+C_{D}C_{f}\right)}{g_{m}C_{f}}\right)}$$

The transimpedance function *H(s)_CSA_* possesses dominant poles at *1/R_f_C_f_* and non-dominant poles at $g_{m}C_{f}/\left(C_{L}C_{D}+C_{L}C_{f}+C_{D}C_{f}\right)$. The high-frequency zeros at *g_m_/C_f_* can be ignored. The approximate value of the *H(s)_CSA_* is equal to *1*/*sC_f_* between the dominant and non-dominant pole frequencies. Therefore, an application with a smaller feedback capacitance may achieve a higher output voltage for a low-input charge. The CSA’s conversion factor can be conducted with the feedback capacitance, whereas the rise time is normally constrained by the non-dominant pole. The rise time *t_r_* of the CSA is further derived:

(9) $$t_{r}=\frac{C_{L}C_{D}/C_{f}+C_{L}+C_{D}}{g_{m}}$$

To keep the non-dominant pole frequency sufficiently high, low input capacitances and load capacitances, as well as high OTA transconductance, are necessary. A moderate increase in *C_f_*, when the charge sensitivity is in the acceptable range, further contributes to reducing the rise time *t_r_*.

The fast rising time of the CSA can reduce the accumulation of pulses at the rising edge, which is conducive to improving the system’s counting capacity. The feedback resistor *R_f_* of the CSA is typically large in order to improve the charge collection efficiency. Nevertheless, the feedback resistor *R_f_* with shunt capacitance *C_f_* results in a long fall time of the CSA output, as shown in Figure 6. In high counting rate applications, pulse pile-up would undoubtedly appear on the falling edge of the CSA output if the interval between two adjacent events was shorter than the fall time. To mitigate pulse pile-up, the CSA is usually followed by a pulse shaper, which can regulate the shape of the output pulse to reduce the fall time of the CSA output. Meanwhile, the smooth pulse generated by the pulse shaper improves the signal-to-noise ratio (SNR) of the analogue front-end chains. This modification facilitates the pulse amplitude measurement because the maximum is gradually rounded instead of steeple-shaped.

**Figure 8:**
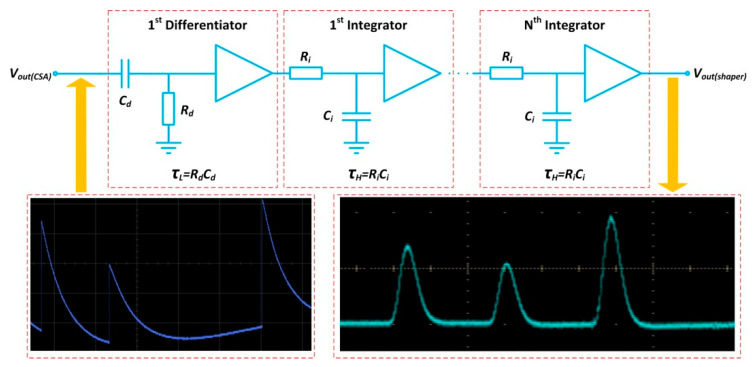
Circuit topology of the pulse shaper. The oscillogram in the red dotted box at the bottom left shows several long-tail pulses from the CSA. Correspondingly, some quasi-Gaussian pulses with the peaking time *τ_p_* in the red dotted box at the bottom right can be generated at the shaper output end.

The pulse shaper consists of a C–R differentiator and an R–C integrator, which form a high-pass filter and a low-pass filter, as shown in Figure 8. If a more symmetrical pulse is required for high SNR applications, the shaper’s order can be increased. As more than one R–C integrator is adopted, a quasi-Gaussian pulse can be generated at the shaper output end, which contains the timing and intensity information.

The transfer function of the pulse shaper is given as follows:

(10) $$H{\left(s\right)}_{shaper}=\left[\frac{s\tau_{H}}{1+s\tau_{H}}\right]{\left[\frac{1}{1+s\tau_{L}}\right]}^{n}$$

where *n* is the number of R–C integrators that corresponds to the pulse shaper’s order, *τ_L_* is the time constant of the differentiator and *τ_H_* is the time constant of the integrator.

The time constant of the differentiator and the time constant of the integrator, taking the schematic parameters in Figure 8 as an example, are given as follows:

(11) $$\tau_{L}=\frac{1}{2\pi f_{L}}=R_{d}C_{d}$$

(12) $$\tau_{H}=\frac{1}{2\pi f_{H}}=R_{i}C_{i}$$

where *f_H_* and *f_L_* are the high cut-off frequency and the low cut-off frequency of the pulse shaper, respectively.

The fall time of the shaped pulse at the output end of the pulse shaper is generally shorter than that of the CSA, which can be regulated by the high cut-off frequency. Similarly, the rise time of the shaped pulse is normally governed by the low cut-off frequency.

Assuming *R_i_C_i_* = *R_d_C_d_*, the correlation between the peaking time *τ_p_* of the shaped pulse and the time constant *τ_shaper_* is given as follows:

(13) $$\tau_{p}=n\tau_{shaper}=nR_{d}C_{d}=nR_{i}C_{i}$$

Increasing the pulse shaper’s order will result in a larger peaking time *τ_p_* for the shaped pulse. The duration of the shaped pulse may exceed the interval between the arrival times of the two continuous photocurrent pulses. In this case, the pulse pile-up will occur again. Therefore, the peaking time needs to be adjusted to avoid pulse overlap as much as possible, and a fast pulse shaper is usually used for high counting rate applications. However, it is arduous to circumvent the pile-up of pulses on account of random pulse intervals. There are always counting errors, and the error of the pulse shaper can also be elucidated using Equation (5).

The entire detection system’s counting error can typically be described by the time factor *τ_total_* [23]. The time factor *τ_total_* is further considered as the cumulative response time contributed by each time constant of the signal pathway. Taking the above analysis as an example, the time factor *τ_total_* can be calculated as:

(14) $$\tau_{total}=\sqrt{\tau_{rd}^{2}+t_{r}^{2}+\tau_{shaper}^{2}}$$

where *τ_rd_* is the release time of the induced charge, *t_r_* is the CSA’s response time and *τ_shaper_* is the time constant of the pulse shaper. The total time constant *τ_total_* characterizes the response speed of the system. The margin of error for a high counting rate system can be further evaluated according to the accuracy requirements of object detection.

## 3. Results and Discussion

**Figure 9:**
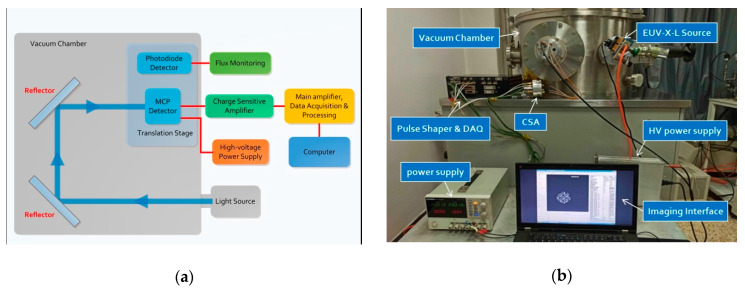
(**a**) Schematic of the experimental setup for the system test. (**b**) Photograph of the laboratory test apparatus. Except for the MCP detector and the photodiode in the vacuum chamber, the rest of the components were placed outside, such as the CSA and the high-voltage power supply. The vacuum chamber was chosen not only for the FUV band but also for a dark background.

This section is divided by subheadings, which provide a concise description of the experimental results, their interpretation and the experimental conclusions. We thoroughly characterized several critical features of the fabricated detection system: the electronic noise, dark background noise, counting linearity and spatial resolution. Figure 9a depicts the experimental block diagram of the system’s characteristic measurement. A photograph of the laboratory test apparatus is shown in Figure 9b. The experimental setup consists of an FUV radiation source, the system to be tested, a high-voltage power supply, 3-axis stages and incident intensity monitoring equipment. Initially, an FUV-X-L flow lamp was used to provide uniform photon flux over the area of detection. The front of the MCP detector was masked with resolution objects, allowing only incident particles to reach the detector surface in the hollowed-out area. A high-voltage power supply provided appropriate biasing for the MCP stack using a resistive divider. The anode signal was passed to an inverting CSA through a vacuum chamber feedthrough. Subsequently, signals were routed to pulse shapers in the main amplifier and finally collected by a computer. In addition, a standard photodiode mounted next to the MCP surface served as an intensity monitor, providing absolute flux estimates. The high-precision microdisplacement control platform enabled alternating measurements between the MCP detector and standard photodiode.

### 3.1. Noise Test

**Figure 10:**
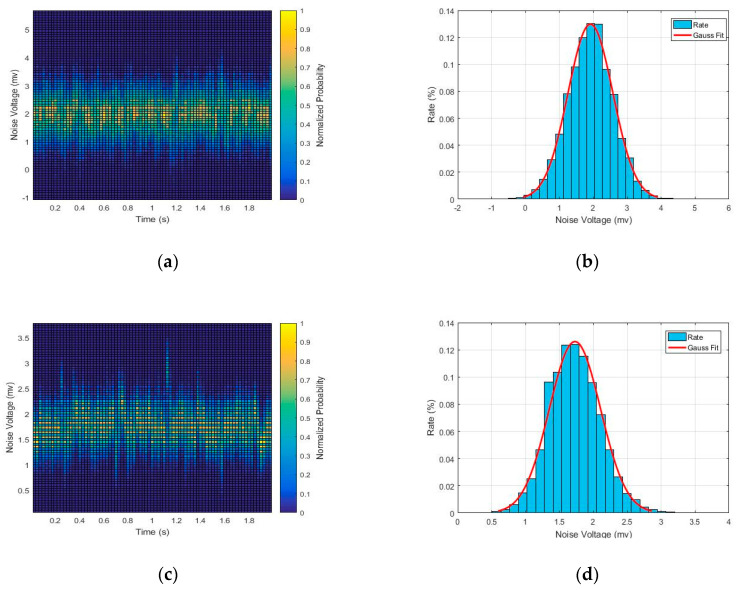
The readout noise of the electronic system: (**a**) the distribution of noise density without the system noise reduction technique; (**b**) the noise histogram and its Gaussian fit for (a); (**c**) the distribution of noise density after the system noise reduction; (**d**) the noise histogram and its Gaussian fit for (c).

The detection system’s noise mainly includes two aspects: the readout noise of the electronic system and the inherent noise of the detector. The readout noise of the electronic system was measured first, ignoring the analogue-to-digital converter (ADC). As illustrated in Figure 10a, the noise voltage at 4 mV peak-to-peak was obtained at the output end of the Gaussian shaper described in Figure 8 without anode capacitance, also known as 0 pF noise. The peak-to-peak value of the noise voltage was reduced to 2.5 mV by optimizing the layout and other noise reduction technologies [24], as shown in Figure 10c. To evaluate the readout noise, histograms at the pedestal are plotted, as shown in Figure 10b,d, with and without the system noise reduction technique, respectively. As shown, the noise voltage presents a Gaussian distribution with a root mean square (RMS) value of approximately 0.6 mV, corresponding to the equivalent input noise charge (EINC) of 352 electrons. The readout noise was notably reduced to 220 electrons after system noise reduction. Gaussian-fitted curves of the noise data are also plotted in Figure 10b,d. Because the MCP gain conforms to a Gaussian distribution, the EINC of the electronic system should be much smaller than the average gain for MCP to ensure that the electronic noise does not affect the counting accuracy and SNR.

**Figure 11:**
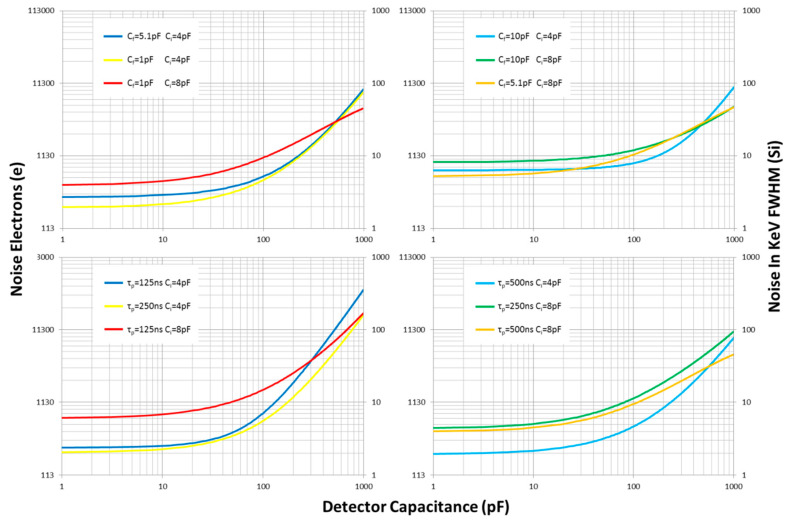
The readout noise as a function of the MCP detector’s capacitance *C_D_*, feedback capacitance *C_f_*, peaking time of the pulse shaper *τ_p_* and input capacitance of readout circuits *C_i_*. The total input capacitance consists of the anode capacitance and the parasitic capacitance at the input terminal.

The readout noise is affected by the feedback capacitance, the time constant of the pulse shaper and the equivalent capacitance of the electronics input end, including the detector’s anode capacitance and the stray capacitance. Further experiments were carried out under these parameters. The data curves presented in Figure 11 are the fitting results obtained with several feedback capacitances and different peaking times of the pulse shaper. The readout noise is non-linear with respect to the detector capacitance *C_D_*, 25 eV/pF at *C_D_* of 30 pF, and 106 eV/pF at *C_D_* of 400 pF. The detector capacitance *C_D_* in total capacitance *C_i_* can be minimized by optimizing the dielectric constant, thickness and insulation channel size of the anode substrate. The stray capacitance *Cs* in the total capacitance *C_i_* can be minimized by optimizing the PCB layout and selecting the appropriate PCB material. Therefore, use of the low-capacitance design for the detector and the reduction technique for the stray capacitance would help to reduce readout noise. For low-capacitance detectors, an input stage with a small *C_i_* should be chosen to make the features of interest stand out. For very high-capacitance detectors, two or more matched input stages with high *C_i_* are usually paralleled to achieve better noise performance. The noise sources also include the feedback resistor *R_f_* of the CSA, the OTA and the pulse shaper. The feedback resistance should be as large as possible to reduce the feedback network’s equivalent parallel current noise. The OTA’s noise can be minimized by selecting an OTA with low voltage noise and current noise on the B input. Care should be taken in selecting an OTA due to the different processing techniques. The resistor’s thermal noise and operational amplifier (OP-AMP) noise can be ignored in the pulse shaper. Increasing the peaking time of the pulse shaper typically improves the symmetry of the output pulse and reduces the noise. In previous work, the long coaxial cable between the detector and the CSA caused the large cable capacitance to contribute to additional noise. The CSA used a field effect transistor (FET) and OP-AMP instead of the OTA. The channel voltage noise and gate current noise of FET are more challenging to improve than the OTA.

**Figure 12:**
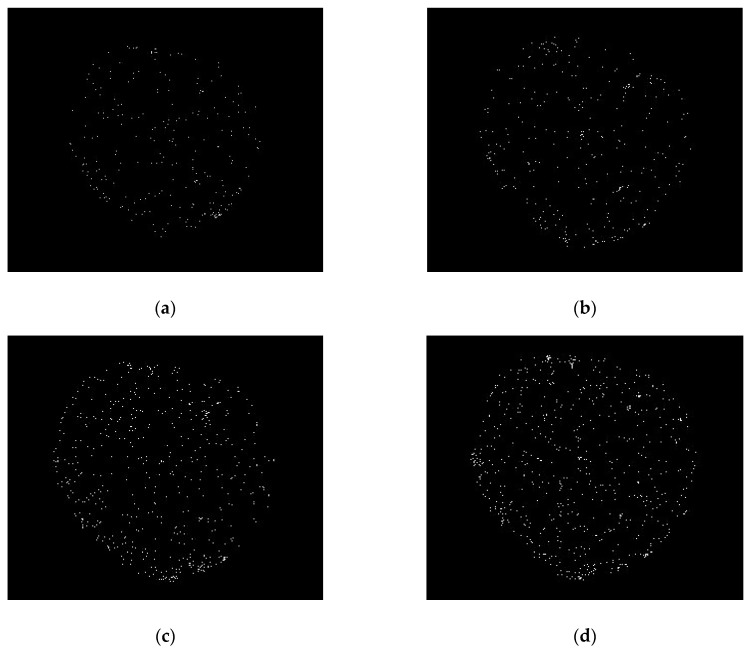
Background noise measured with the incident source removed under different ambient temperature conditions (*T_A_*). Long collection times, such as 120 s, were selected to avoid random error in the experiment: (**a**) *T_A_* = 300 K, *N_D_* = 0.610 c/(s cm^2^); (**b**) *T_A_* = 310 K, *N_D_* = 0.770 c/(s cm^2^); (**c**) *T_A_* = 320 K, *N_D_* = 1.405 c/(s cm^2^); (**d**) *T_A_* = 330 K, *N_D_* = 1.793 c/(s cm^2^).

The detection system’s dark background noise is defined as the counts per unit time per unit area in the absence of incident photons, which determines the lower limit of the dynamic range for the detection system. The dark counts arise mostly from charge pulses produced by the MCP detector when the readout system’s noise is low enough to be ignored. They typically originate from the photocathode’s thermal electron emission, the field emission on the MCP surface when charged up, the ion feedback caused by residual gas in MCP and the radioactive decay of ^40^K and ^87^Rb in the glass of MCP. The change in ambient temperature is a pivotal factor affecting the dark count of the MCP detector. The distribution of dark background events at different ambient temperatures is shown in Figure 12. The dark count rates (*N_D_*) increase with temperature, which are 0.610 c/(s cm^2^) at 300 K and 1.793 c/(s cm^2^) at 330 K, almost three times the rate at room temperature. The MCP detector is more sensitive to ambient temperature than the electronic components. As a result, there is a favorable scheme for thermal control in orbit, which is controlled at the objective value of 290 K ± 5 K [25].

**Figure 13:**
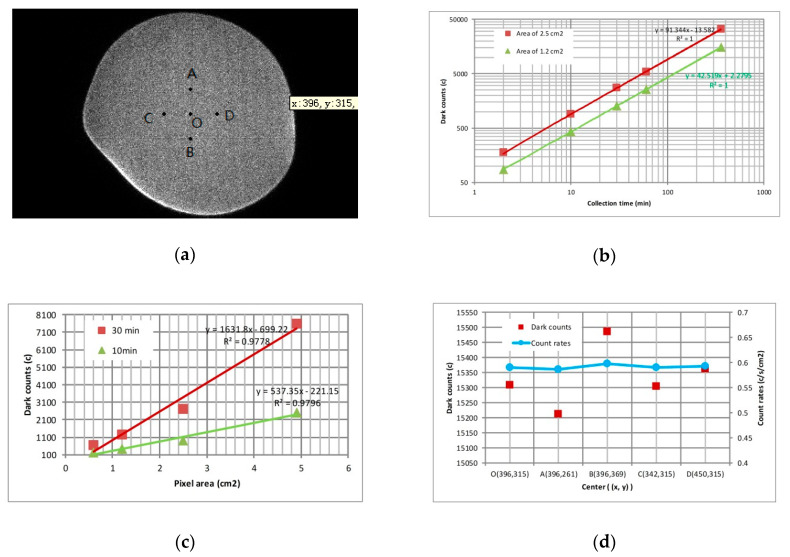
Statistical characteristics of dark counts: (**a**) continuous collection for 360 min on a dark background; (**b**) dark counts variance over collection time; (**c**) dark counts changes with different pixel areas; (**d**) dark counts in the same area with different center points. The selected area should avoid the edge of the image because the edge is distorted.

To calculate the distribution characteristics of dark background noise, an experiment with a maximum collection time of 360 min was carried out. As shown in Figure 13a, the dark noise image is even and smooth. There are no bright spots in the center of the image, which is due to debris on the MCP surface and the memory effect of the MCP detector. As shown in Figure 13b, the dark counts for the detection system increase linearly with the collection time under the same area. Furthermore, the dark counts were calculated by intercepting different pixel areas when a specific test was completed. The calculation result shows that the dark counts also increase linearly with the pixel area. However, the dark counts will increase non-linearly if the selected area includes the image’s edge, such as the pixel area of 4.9 cm^2^ in Figure 13c. We took points A, B, C, D and O as the center of the circle to intercept the area of 1.2 cm^2^, as shown in Figure 13a, and to calculate the dark counts in the area. Figure 13d plots the fluctuation of dark counts and dark count rates, which is less than 2%. The results show that the long time observation is consistent with the uniform distribution, although the dark background noise is random.

### 3.2. Characterization of Counting Rate Linearity

Count values can always be obtained accurately by using an ideal system with a wide dynamic range, whether the incident intensity is high or low. Unfortunately, compared with the MCP detector, the readout circuits’ responses are commonly the primary factors that limit the counting capacity for the photon counting imaging system in aerospace applications. In fact, the dynamic range of the system will be wider for the same detector if the circuits possess stronger readout capabilities. Because of the randomness of the photon events’ arrival times according to the Poisson distribution, the counting accuracy related to the pulse pile-up is still a problem. To correct the counting error effectively, it is necessary to calibrate the linearity of the system counting rate by adjusting the radiation source’s incident intensity. This calibration experiment was carried out to obtain the actual incident intensity during on-orbit mission operations through test data fitting. The experimental device is shown in Figure 9. The MCP detector to be tested and the standard photodiode detector are installed on the 3-axis stages. A narrow band filter was placed between the source and the detector to estimate the counting rate according to the standard detector output value.

**Figure 14:**
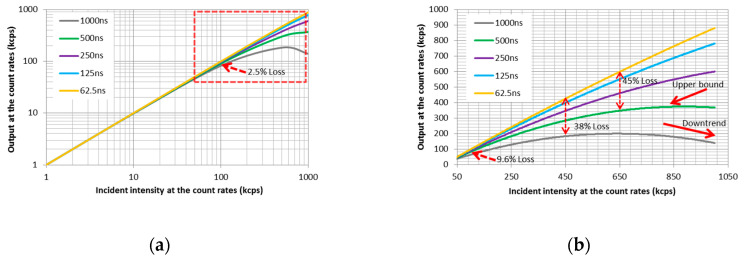
The corresponding curves of the counting rate at the output end with the changing incident intensity. The system counting error is not apparent when the incident intensity is below 50 kcps, regardless of whether the pulse shaper’s peaking time is 62.5 ns or 1000 ns. Nevertheless, the counting error gradually appears with increasing incident photon rates, as shown in the red dotted box in (**a**). More details of the curves in this area are shown in (**b**).

Figure 14 depicts the counting rate of the system output as a function of the incident intensity for different peaking times of the pulse shaper, which characterizes the detection system’s linearity. The abscissa value represents the actual photon counting rate corresponding to the photocurrent obtained at the standard photodiode’s output. The ordinate value shows the photon counting rate collected by the detection system that is to be measured. When the radiation intensity is weak, the photon counting rate finally acquired by the detection system is approximately linear. In contrast, the non-linearity of the system becomes apparent with increasing radiation intensity.

As illustrated in Figure 14, the deviation of the counting rate due to linearity varies with different peaking times of the pulse shaper. Provided that the incident intensity shown as the electric current is 1 µA, corresponding to 100 kcps, the counting rate at the output end of the system will reach 97.5 kcps with counting losses of 2.5% at a peaking time of 125 ns. The counting rate will decrease to 90.4 kcps with counting losses of 9.6% at a peaking time of 500 ns. Likewise, counting losses of 38% and 45% occur at the incident intensities of 450 kcps and 650 kcps, respectively, as shown in Figure 14b. When the incident intensity is further increased to 770 kcps, the counting rate with a peaking time of 500 ns almost reaches the upper bound of the system capacity. As the incident intensity continues to increase, the photon counting rate collected by this system presents a downward trend at the end of the curve shown in Figure 14b. This phenomenon occurs as a result of multiple pulse superpositions, which the discriminator cannot distinguish accurately.

### 3.3. Spatial Resolution

**Figure 15:**
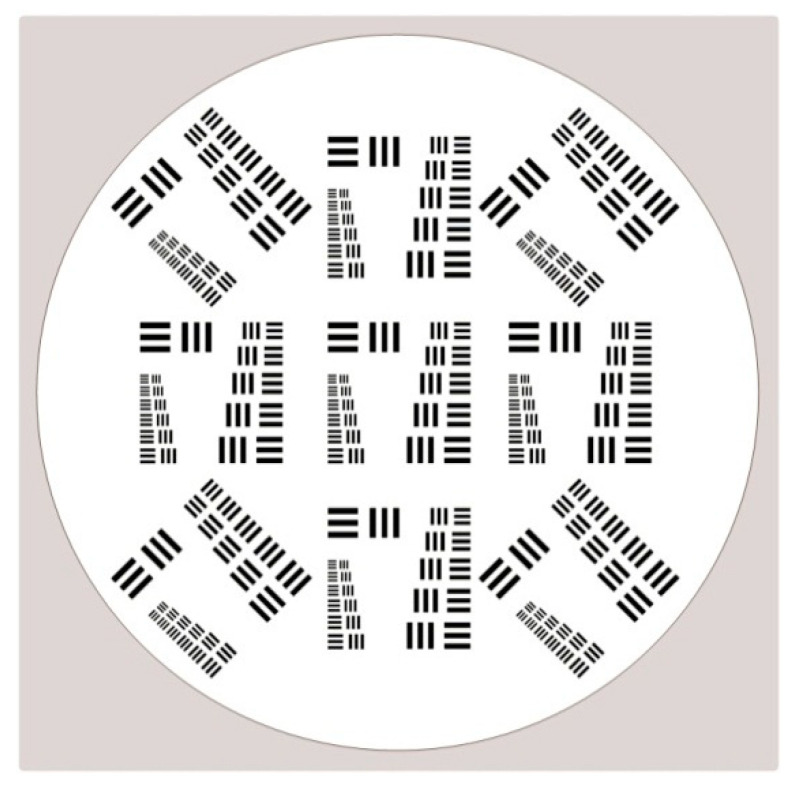
The pattern for the resolution mask used for the experiment. The nine modules are distributed in graphic arrays. Each module contains line pairs with group numbers 1 and 2.

Using counting rates ranging from low to high, corresponding to incident intensities over a wide range, the detection system’s spatial resolution was measured using a resolution mask manufactured by our team according to the MIL-STD-150A format [26]. Figure 15 shows that the pattern consists of groups of three bars with dimensions ranging from large to small. Each group consists of six elements labelled from 1 to 6, and each element is further made up of six bars with equal spacing horizontally and vertically. In fact, the largest bar the detection system cannot discern is commonly defined as the limitation of its resolving power. To determine the spatial resolution, the resolution mask was mounted directly on the detector surface and a uniform source of radiation was placed in front of the resolution mask. The detector’s imaging was performed while the incident intensity with different counting rates was adjusted through several attenuating filters. Considering that the distance from the radiation source to the detector was much larger than the distance between the resolution mask and the detector, the effect of the optical expansion of the mask aperture was small enough to be ignored.

**Figure 16:**
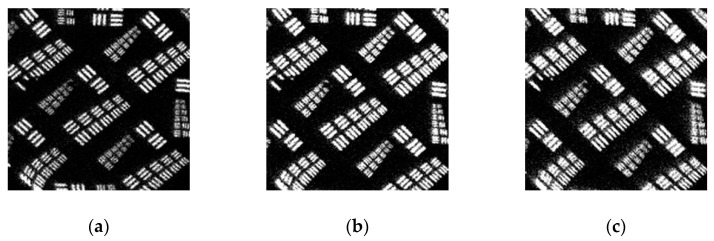

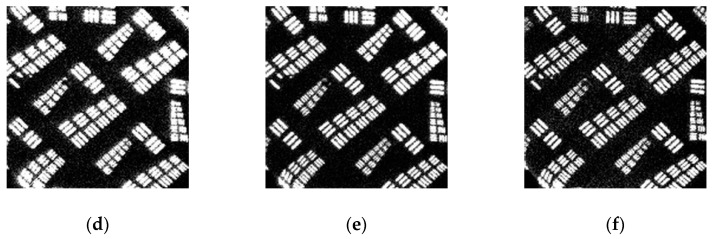
Imaging of the resolution mask with different incident intensities (*I_in_*) and peaking times for the pulse shaper (*T_p_*). The edges of the images are slightly distorted due to the mutual capacitance between anode electrodes: (**a**) *I_in_* = 100 kcps, *T_p_* = 500 ns; (**b**) *I_in_* = 200 kcps, *T_p_* = 500 ns; (**c**) *I_in_* = 300 kcps, *T_p_* = 500 ns; (**d**) *I_in_* = 400 kcps, *T_p_* = 500 ns; (**e**) *I_in_* = 400 kcps, *T_p_* = 200 ns; (**f**) *I_in_* = 400 kcps, *T_p_* = 100 ns.

The resolution results for the whole plane at specific incident event rates are shown in Figure 16. The spatial resolution is usually better in the central region of the detector than at the edge. The edge probably exhibits some distortions due to the edge field effect of MCP and the pattern of a wedge strip anode. For a high-precision measurement system, the central region should be used first because it can obtain clear images in the entire imaging range. Figure 16 presents six images at different counting rates and peaking times of the pulse shaper taken with the detector in the image charge configuration. As illustrated in Figure 16a,b, the spatial resolution can exceed 125 µm in the horizontal and vertical directions, corresponding to group two’s first element in the resolution mask. By contrast, at a higher counting rate of 300 kcps, the spatial resolution in the horizontal direction decreases to 140 µm in Figure 16c, corresponding to the sixth element in group one. From the resolution mask image, the detector image was slightly blurred but in working order. However, the system’s image quality was significantly degraded, as shown in Figure 16d, when the light intensity continued to increase to 400 kcps. The piled-up pulse introduces error into the charge pulse amplitude measurement, which becomes a noise spot for the reconstructed image, thus deteriorating the spatial resolution. This phenomenon is typically caused by the fact that the readout circuits’ response times are greater than the pulse interval. As a result, the peaking time of the pulse shaper can be reduced to avoid the accumulation of the tail of the pulse, which improves the spatial resolution to 111 µm, as shown in Figure 16e,f.

**Figure 17:**
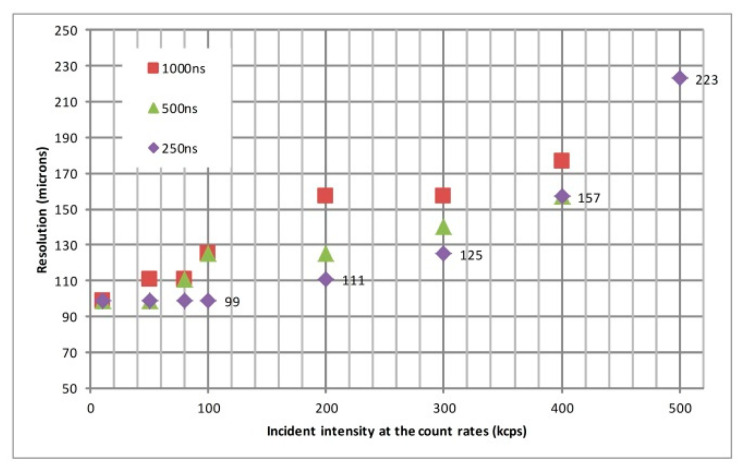
The image resolution plot versus incident photon counting rate with different peaking times of the pulse shaper. The resolution value that can be achieved by the system applies a fixed scale from the 1951 USAF resolution mask. For a specific peaking time of the pulse shaper, the resolution will decrease rapidly with a massive increase in incident intensity.

Figure 17 plots the resolution versus the incident photon counting rate, showing data taken at different pulse shaper peaking times. For an incident photon counting rate of 50 kcps, there is no significant difference in resolution between the peaking times of 250 and 500 ns. A higher SNR can be more easily obtained with a longer peaking time. If peaking time is not dominant in the pile-up, the resolution with a longer peaking time is slightly better than that with a shorter peaking time at a low count rate. Nevertheless, with increasing incident photon rates at 200 or 300 kcps, the probability of pulse pile-up at the peaking time of 250 ns is less than the peaking time of 1000 ns. The peaking time of 250 ns exhibits significantly better spatial resolution than the peaking time of 1000 ns. Furthermore, at a higher counting rate, the resolution begins to decrease, although the pulse shaper’s peaking time is adjusted. The pulse pile-up is not only dominated by peaking time, but also related to the response time of the CSA. The CSA saturation would also be a consideration, since the pile-up of incident events may exceed the dynamic range of the CSA, resulting in resolution degradation.

**Figure 18:**
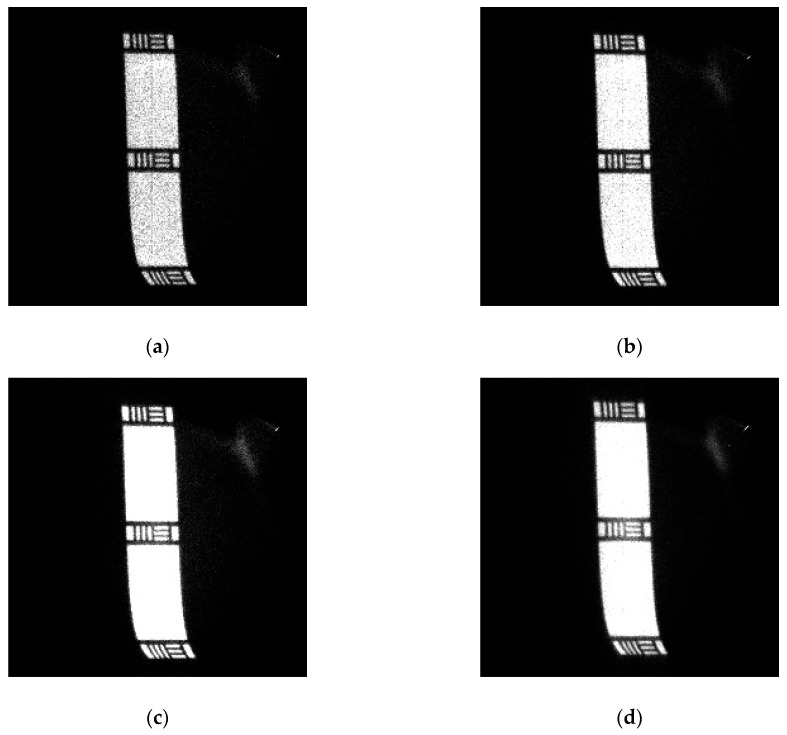
Special pattern imaging to verify locally high incident intensity: (**a**) incident intensity at 200 kcps; (**b**) incident intensity at 300 kcps; (**c**) incident intensity at 400 kcps; (**d**) incident intensity at 500 kcps.

The localized resolution of the detection system is crucial when the distribution of the object detection in the field of view is extremely non-uniform. Thus, a special pattern mask was manufactured to receive incident light for further tests. As shown in Figure 18, the format was divided into two parts: a rectangular area for light transmission and a resolution area for a 250 µm line pair. The mask simulated the inhomogeneity of space objects by varying the number of photons reaching the MCP surface. The number of incident photons for electron multiplication in the rectangular area accounted for most of the total photons. The resolution images of the 250 µm line pair were acquired individually when the uniform light intensity changed from 200 to 500 kcps. The results show that the 250 µm line pairs can be clearly distinguished in four images. If the gain degradation from the local event saturation rate in the MCP detector is not obvious and the release time of the induced charge between the germanium film and the anode is carefully selected, it will not decrease the spatial resolving power at a local high count rate.

### 3.4. Product Performance

The photon counting image system was applied to a WAI instrument for the FY3 satellite mission. The detailed ground tests and calibration experiments were carried out before the launch. See [8] for more details. The WAI instrument parameters are summarized in Table 1 to enable comparisons with the test indexes of the EUVC instrument developed previously by our team [6,8]. The indexes of sensitivity, dark counts and maximum counting rates for the WAI are better than those for the EUVC. Low dark counts are among the necessary conditions for high sensitivity. If the sensitivity meets the mission requirements, the angular resolution and counting rates should be better considered. As the focal length of WAI is 18.5 mm, the spatial resolution of the detection system discussed in this paper corresponds to an angular resolution of ~0.29°, which is better than the mission requirement of 0.8°. Under the circumstances, the WAI’s angular resolution depends on the optical system rather than the photon counting imaging system. Therefore, the readout electronics, such as the CSA and pulse shaper, are key bottlenecks that affect the particular mission’s counting rate. The maximum counting rate is actually designed to be 350 kcps according to the requirements.

## 4. Conclusions

This paper has detailed an FUV photon counting image system using the MCP detector and compact electronics. Photon streams from several photons to tens of thousands in a second for an intensity object are thoroughly detectable and can be discriminated with low or zero error rates. This measuring instrument with a wide dynamic range is particularly necessary for aerospace applications, where extreme conditions such as strong or weak light need to be taken into account. Admittedly, MCP detectors with a chevron stack appear to have a very fast response. However, the charge induction readout methods must be carefully considered to obtain the optimal time characteristic. Moreover, the readout circuits’ response time requires more attention, as it is also related to pulse pile-up. There is a trade-off between noise and response time in the readout circuits, but the response time is generally more important because the counting rate plays a crucial role when the light intensity is of large magnitude. Additionally, a slight increase in noise usually has no significant impact on imaging quality.

Future studies will further develop this fast readout technology to accommodate the projected detector count rate. Although the analogue peak holder has proven propitious for sensitive detection in flight instruments, the peak holder’s charge and discharge time characteristics are still critical considerations. These issues must be circumvented to improve the counting capacity of detection systems. In addition, the latest high-speed acquisition technology and specific algorithms might be considered for the peak reconstruction of shaped pulses. Pulse shapers can also be implemented using more sophisticated digital filtering technology that is more flexible and efficient. A new aurora imaging instrument based on the above methods is currently under consideration for the next generation of Fengyun satellites.

## Figures and Tables

**Table 1:** Test Results of WAI and EUVC.

Index	WAI	EUVC
Field of view	134.43° × 133.24°	14.7° (Circular)
Angular resolution	0.34°	0.08°
Sensitivity	8.8 count s^−1^ Rayleigh^−1^	0.11 count s^−1^ Rayleigh^−1^
Dark counts	0.610 counts s^−1^ cm^−2^	1.000 counts s^−1^ cm^−2^
Maximum counting rate	350 kcps	66 kcps

## Abbreviations

The following abbreviations are used in this manuscript:

FUV	Far-Ultraviolet
MCP	Microchannel Plate
WSA	Wedge Strip Anode
CSA	Charge Sensitive Amplifier
EUV	Extreme Ultraviolet Camera
WAI	Wide-angle Aurora Imager
LBH	Lyman–Birge–Hopfield
FWHM	Full-Width Half-Maximum
ASIC	Application Specific Integrated Circuit
FPGA	Field Programmable Gate Array
OTA	Operational Transconductance Amplifier
OP-AMP	Operational Amplifier
FET	Field Effect Transistor
SNR	Signal-to-Noise Ratio
DAQ	Data Acquisition
ADC	Analog-to-Digital Converter
EINC	Equivalent Input Noise Charge
